# Bundling of collagen fibrils influences osteocyte network formation during bone modeling

**DOI:** 10.1038/s41598-023-48786-y

**Published:** 2023-12-12

**Authors:** Mana Hashimoto, Haruka Takahashi, Kaori Tabata-Okubo, Noriyuki Nagaoka, Kazuaki Tokunaga, Haruka Matsumori, Yoshihito Ishihara, Masaru Kaku, Tadahiro Iimura, Toru Hara, Hiroshi Kamioka

**Affiliations:** 1 Department of Orthodontics, Graduate School of Medicine, Dentistry and Pharmaceutical Sciences, Okayama University, 2-5-1, Shikata-cho, Kita-Ku, Okayama, Okayama 700-8525 Japan; 2 Department of Orthodontics, Okayama University Hospital, 2-5-1, Shikata-cho, Kita-Ku, Okayama, Okayama 700-8525 Japan; 3 Advanced Research Center for Oral and Craniofacial Sciences, Okayama University Dental School, 2-5-1, Shikata-cho, Kita-Ku, Okayama, Okayama 700-8525 Japan; 4 Nikon Corporation, 2-15-3 Konan, Minato-Ku, Tokyo, 108-6290 Japan; 5 Division of Bio-prosthodontics, Faculty of Dentistry and Graduate School of Medical and Dental Sciences, Niigata University, 2-5274 Gakkocho-dori, Chuo-Ku, Niigata, Niigata 951-8514 Japan; 6 Department of Pharmacology, Faculty and Graduate School of Dental Medicine, Hokkaido University, N13 W7, Kita-Ku, Sapporo, Hokkaido 060-8586 Japan; 7 Research Center for Structural Materials, National Institute for Materials Science, 1-2-1, Sengen, Tsukuba, Ibaraki 305-0047 Japan

**Keywords:** Cell biology, Structural biology

## Abstract

Osteocytes form a cellular network by gap junctions between their cell processes. This network is important since intercellular communication via the network is essential for bone metabolism. However, the factors that influence the formation of this osteocyte network remain unknown. As the early stage of osteocyte network formation occurs on the bone surface, we observed a newly formed trabecular bone surface by orthogonal focused ion beam-scanning electron microscopy. The embedding late osteoblast processes tended to avoid bundled collagen fibrils and elongate into sparse collagen fibrils. Then, we examined whether the inhibition of bundling of collagen fibrils using a potent lysyl oxidase inhibitor, β-aminopropionitrile (BAPN) changed the cellular network of the chick calvaria. The osteocyte shape of the control group was spindle-shape, while that of the BAPN group was sphere-shaped. In addition, the osteocyte processes of the control group were elongated vertically to the long axis of the cell body, whereas the osteocyte processes of the BAPN group were elongated radially. Therefore, it was suggested that the bundling of collagen fibrils influences normal osteocyte network formation during bone modeling.

## Introduction

Osteocytes, which are mechanosensory cells of bone, represent 90–95% of the cells in bone^1–4^. Previous reports have shown that osteocytes are deeply involved in bone homeostasis by controlling bone mineralization; thus, they have received a great deal of attention^5–8^. Osteocytes form a cellular network by gap junctions between their cell processes^9,10^. This osteocyte network is of great importance since intercellular communication via the osteocyte network is essential for the control of bone homeostasis^11^. We previously reported that the osteocyte network becomes progressively more regular with the application of mechanical stress during skeletal growth and remodeling^12^. However, the factors that influence osteocyte network formation during bone modeling and early bone formation remain unknown.

The maturation process of the bone matrix starts with the release of collagen fibrils from osteoblasts. Thereafter, collagen fibrils bundle to form collagen fibers. The bone increases its thickness as a matrix through repeated collagen fiber formation. Hydroxyapatite is deposited into collagen fibers, where they form calcified nodules. On coming into contact with collagen fibers, they calcify an expansive matrix of collagen. The calcification spreads with the collagen acting as a scaffold^13,14^. The bone matrix then becomes stiff while maintaining flexibility. However, osteoblasts become osteocytes in the process of increasing the thickness of the bone matrix. The osteocyte network becomes more difficult to reconstruct as the bone matrix hardens. We previously reported that the shape of the intracanalicular surface is dependent on the running of collagen fibrils^15^. The osteocyte processes and the collagen fibrils may be related to each other. Therefore, the purpose of this study was to investigate whether the osteocyte network in embryonic chick calvaria is affected by the maturation process of the bone matrix, especially the bundling of collagen fibrils.

We used the embryonic chick calvaria in this study because it grows from the orbital side to the parietal side, and the definition of the long axis of the trabecular bone is relatively easy^16,17^. As the early stage of osteocyte network formation occurs on the bone surface, we observed the newly formed trabecular bone surface and tip area of trabecular bone in the embryonic chick calvaria in this study.

We observed the positional relationship between the bundled collagen fibrils (collagen fibers) and the embedding late osteoblast processes by three-dimensional reconstruction with orthogonal focused ion beam–scanning electron microscopy (FIB–SEM) and the Amira software program as previously reported^18^. FIB–SEM can produce three-dimensional reconstructed images with a resolution of several nanometers, as the surface of the bone tissue is cut by the FIB. Accordingly, we can observe specimens in a wide range of sizes, from individual nanosized collagen fibrils to microsized bone cells.

Lysyl oxidase (LOX) is essential to the initiation of collagen cross-linking^19,20^. We used an LOX inhibitor, β-aminopropionitrile (BAPN) to inhibit the bundling of collagen fibrils (collagen fibers)^20,21^. Second harmonic generation (SHG) images of the collagen fibrils were observed by multiphoton laser microscopy, and we analyzed the influence of BAPN on bundling of the collagen fibrils. The morphologic change of the osteocytes was measured by confocal laser scanning microscopy (CLS) and the Amira software program. We observed, for the first time, osteocyte network formation when the bundling of collagen fibrils was inhibited. As a result, it was suggested that the bundling of collagen fibrils influences normal osteocyte network formation during bone modeling.

## Results

**Figure 1:**
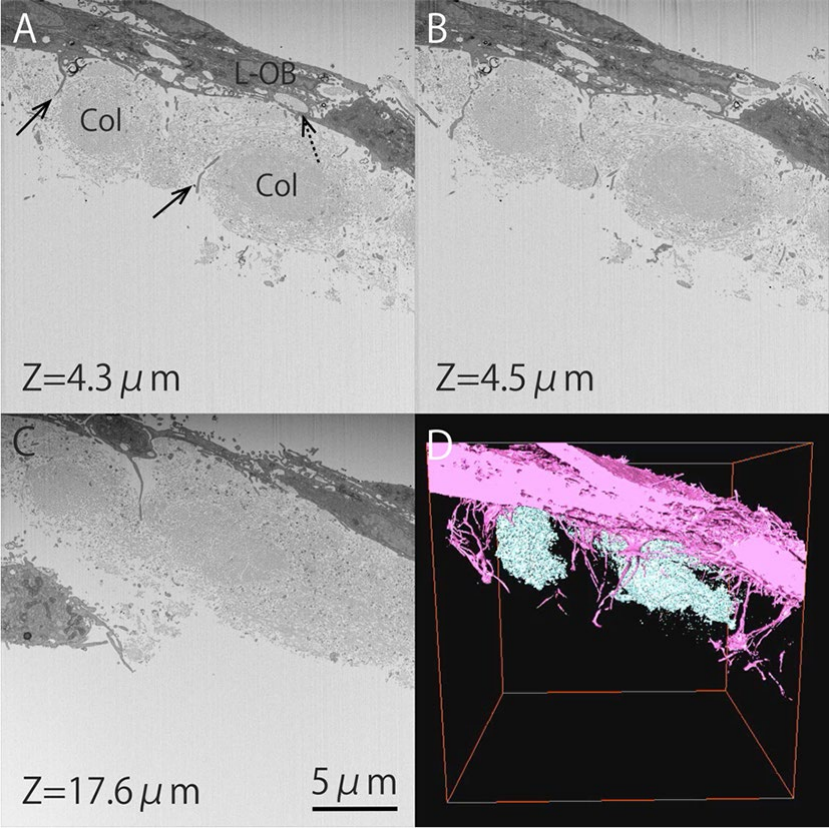
Observation of the trabecular bone by orthogonal FIB–SEM. (**A**–**C**) Serial images (X–Y plane) observed by orthogonal FIB–SEM. L-OB, embedding late osteoblast; Col, collagen fibers. Black solid arrows show embedding late osteoblast processes. The black dotted arrow shows that collagen fibrils are produced from osteoblasts. Scale bar, 5 µm. (**D**) A three-dimensional reconstructed image obtained by orthogonal FIB–SEM. The pink colored area shows the embedding late osteoblasts and their processes. The blue-colored area shows the bundled collagen fibrils (collagen fibers).

Figure 1A–C shows the orthogonal FIB–SEM images of the 25 × 25-µm area. These images show the area of the tip of a single trabecular bone in the embryonic chick calvaria; the resolution was 25 nm/pixel. Serial images were taken along the longitudinal axis of the trabecular bone. One thousand serial images were captured with the sectioning pitch set at 25 nm from the parietal side to the orbital side of the calvaria. Thus, a volume of 25 × 25 × 25 µm was observed for a single trabecular bone (Fig. 1D and Supplementary Video 1).

The lateral direction of the SEM images was defined as the X axis, the vertical direction was defined as the Y axis, and the longitudinal axis of the trabecular bone was defined as the Z axis. The SEM images were shown in reversed black and white to facilitate observation and then converted to TEM-like images.

Figure 1A–C shows the images of the X–Y plane. Embedding late osteoblasts were observed on the surface of the bone (Fig. 1A: L-OB). Bundled collagen fibrils (collagen fibers) were also observed in the center of the trabecular bone (Fig. 1A: Col). The embedding late osteoblast processes elongated into the bone matrix (Fig. 1A, black solid arrows). The collagen fibrils produced from the osteoblasts were observed as previously described (Fig. 1A, black dotted arrow)^16^. The slice pitch is 25 nm, which is thinner than the 100 nm diameter of collagen fibrils, so if collagen fibrils were connected with the osteoblast in these sectional images, we conclude that the collagen fibrils were truly produced from the osteoblast.

Sequentially, the embedding late osteoblasts and collagen fibers were extracted three-dimensionally (Fig. 1D). The pink-colored area shows the embedding late osteoblasts and their processes. The blue-colored area shows the bundled collagen fibrils (collagen fibers). Figure 1D shows that the cell processes tend to avoid bundled collagen fibrils as they elongated into the bone matrix (Supplementary Video 2). Therefore, we thought that osteocyte network formation would be influenced by the bundling of collagen fibrils. We next studied whether inhibiting the bundling of collagen fibrils results in any difference in osteocyte network formation.

BAPN was used to inhibit collagen cross-linking^20,22^. BAPN is an inhibitor of LOX, which is essential for the initiation of collagen cross-linking^19,20^. BAPN was injected into 16-day-old embryonic chicks, after which 19-day-old embryonic chick calvaria were collected.

**Figure 2:**
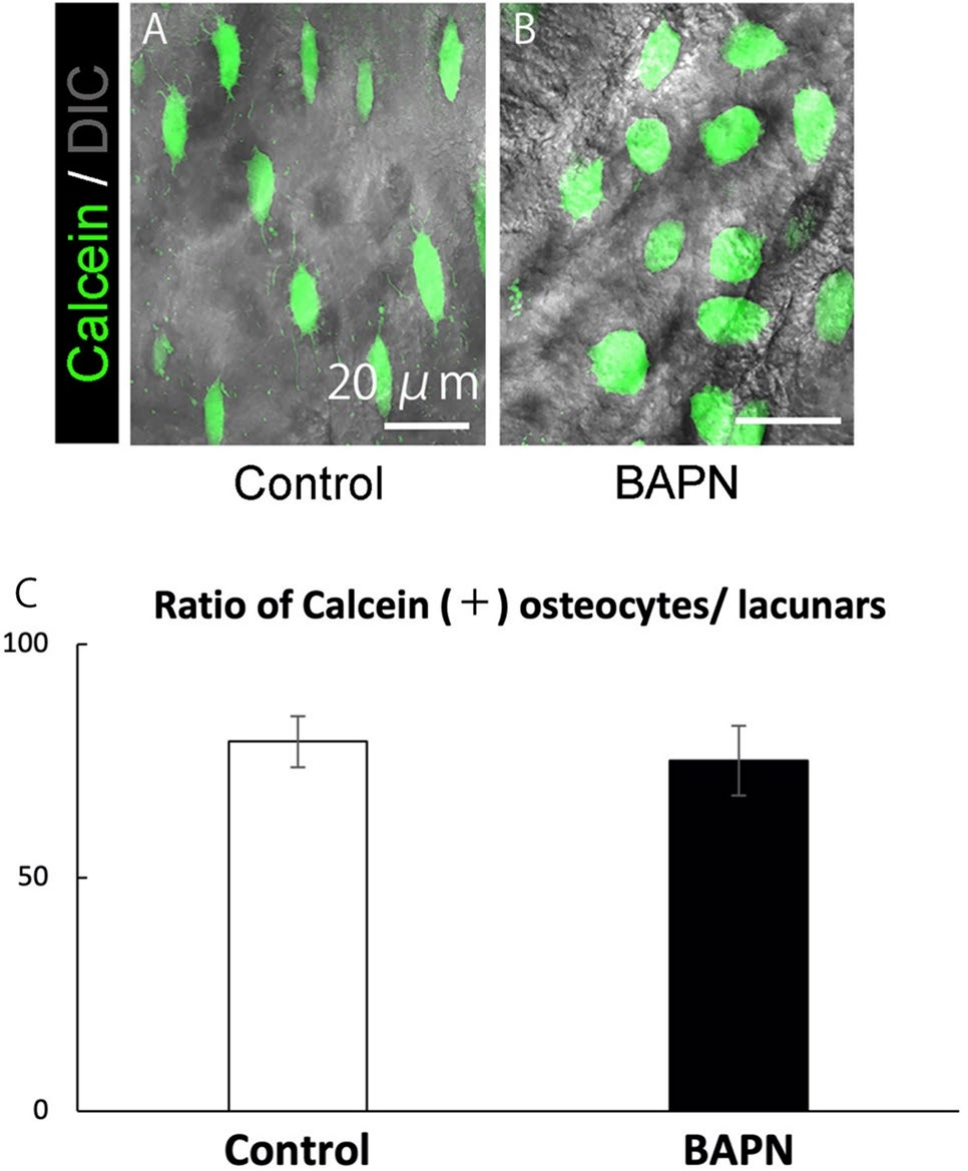
Confirmation of the effect of BAPN on osteocytes. (**A**) Representative fluorescence images of living osteocytes of the control group labeled with calcein-AM in the chick calvaria. Scale bar, 20 µm. (**B**) Representative fluorescence images of living osteocytes of the BAPN group labeled with calcein-AM in the chick calvaria. Scale bar, 20 µm. (**C**) Quantification of osteocyte viability tests. The histogram shows the mean values for the percentage of calcein-AM-positive osteocytes in the lacunae, and the error bars represent the standard deviation. No significant difference was observed (*P* < 0.05).

The ratio of calcein acetoxymethyl ester (calcein-AM)-positive osteocytes to lacunae was measured to evaluate whether BAPN injection influences osteocyte viability. Fluorescence and DIC images exhibited intense green fluorescence in osteocytes within lacunae in both groups (Fig. 2A: control group, Fig. 2B: BAPN group). There were 475 lacunae in the control group, of which 383 were calcein-AM-positive osteocytes, with an average positive rate of 79.24% (SD = 5.49). There were 435 lacunae in the BAPN group, of which 333 were calcein-AM-positive osteocytes, with an average positive rate of 75.18% (SD = 7.46). Quantification results also showed no differences in the percentages of calcein-AM-positive osteocytes between the control and BAPN groups, indicating that BAPN injection into chicks did not affect osteocyte viability in this study (Fig. 2C).

**Figure 3:**
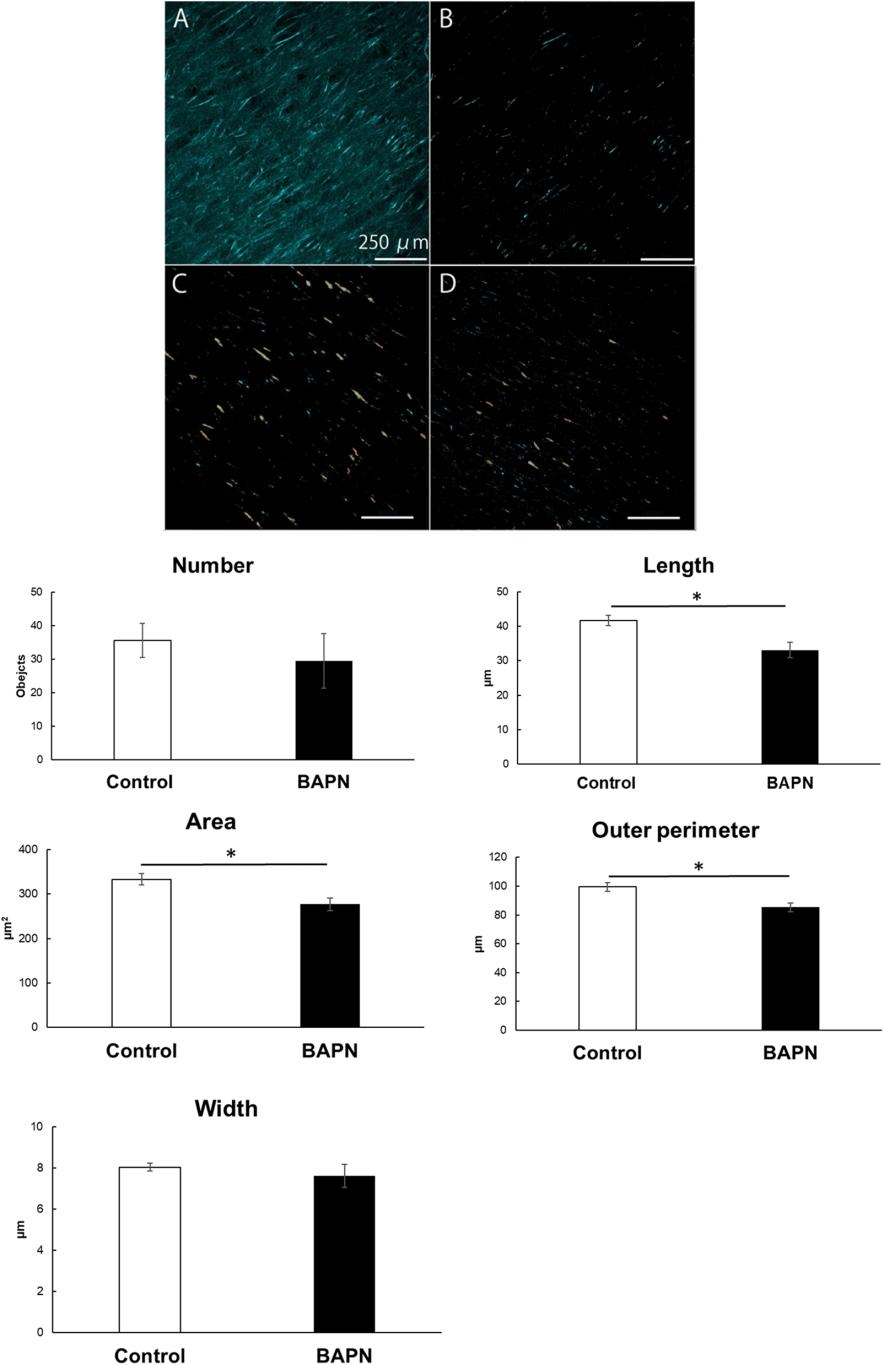
Confirmation that BAPN inhibits collagen fiber formation. (**A)** Representative SHG image of the control group. Scale bar, 250 µm. (**B**) Binarized image of Fig. 3A. Scale bar, 250 µm. (**C**) Representative binarized image with selected geometric parameters of control group. Scale bar, 250 µm. (**D**) Representative binarized image with selected geometric parameters of BAPN group. Scale bar, 250 µm.The SHG images of the collagen fibers were binarized, and their geometric parameters were quantitatively analyzed. The histogram shows each mean value, and the error bars represent the standard deviation. Asterisk indicates a statistically significant difference (*P* < 0.05).

We performed SHG observation with a multiphoton excitation fluorescence microscope. SHG enabled us to visualize fibers or bundles of collagen in bone tissues. Figure 3A shows the representative SHG image of the control group and Fig. 3B shows the binarized image of Fig. 3A. Figure 3C and D show the images which were selected geometric parameters after binarization (Fig. 3C: control group, Fig. 3D: BAPN group). The graphs of Fig. 3 show the quantitative analysis results of the geometric parameters of the collagen fiber binarized images. There was no significant difference in the number or width of the SHG signal between the control and BAPN groups. On the other hand, the length, area and outer perimeter of the BAPN group were significantly smaller than those of the control group. These results indicated that BAPN injection produced collagen fibers with poor continuity.

**Figure 4:**
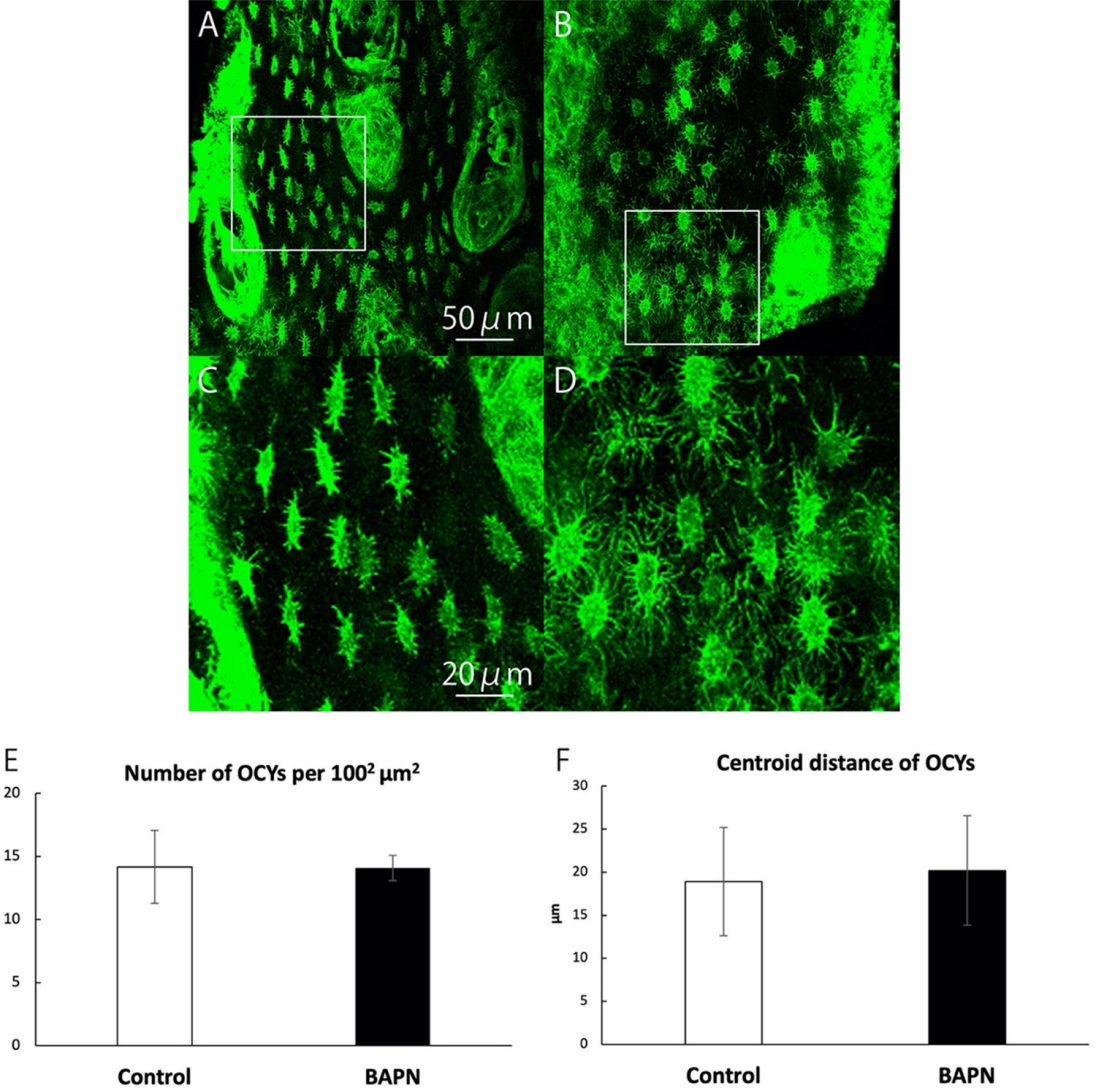
CLS images of osteocytes. (**A**) Image of osteocytes of the control group approximately 3.6 mm from the parietal side. Scale bar, 50 µm. (**B**) Image of osteocytes of the BAPN group approximately 3.6 mm from the parietal side. (**C**) Enlarged view of the white solid frame in Fig. 4A. Scale bar, 20 µm. (**D**) Enlarged view of the white solid frame in Fig. 4B. (**E)** Quantification of the number of osteocytes per 100^2^ µm^2^. The histogram shows the mean number of osteocytes, and error bars represent the standard deviation. There was no significant difference (*P* < 0.05). (**F**) Quantification results of the centroid distance of osteocytes. The histogram shows the mean centroid distance, and error bars represent the standard deviation. There was no significant difference (*P* < 0.05).

Next, osteocytes were stained with phalloidin and observed, from the parietal side to the orbital side of the calvaria, by CLS. Figure 4A and B show osteocyte images at a position approximately 3.6 mm from the parietal side (Fig. 4A: control group, Fig. 4B: BAPN group). Figure 4C shows an enlarged view of the white solid frame in Fig. 4A. Figure 4D shows an enlarged view of the white solid frame in Fig. 4B. The osteocytes of the control group were spindle shaped, while those of the BAPN group were sphere-shaped. The total number of osteocytes in both groups was measured using the Amira software program. The average number of osteocytes per 100 µm^2^ was 14.17 (SD = 2.89) in the control group and 14.10 (SD = 0.99) in the BAPN group, which did not amount to a significant difference (Fig. 4E). The centroid distance of osteocytes was 18.90 (SD = 6.29) µm in the control group and 20.20 (SD = 6.38) µm in the BAPN group, and did not amount to a significant difference (Fig. 4F).

**Figure 5:**
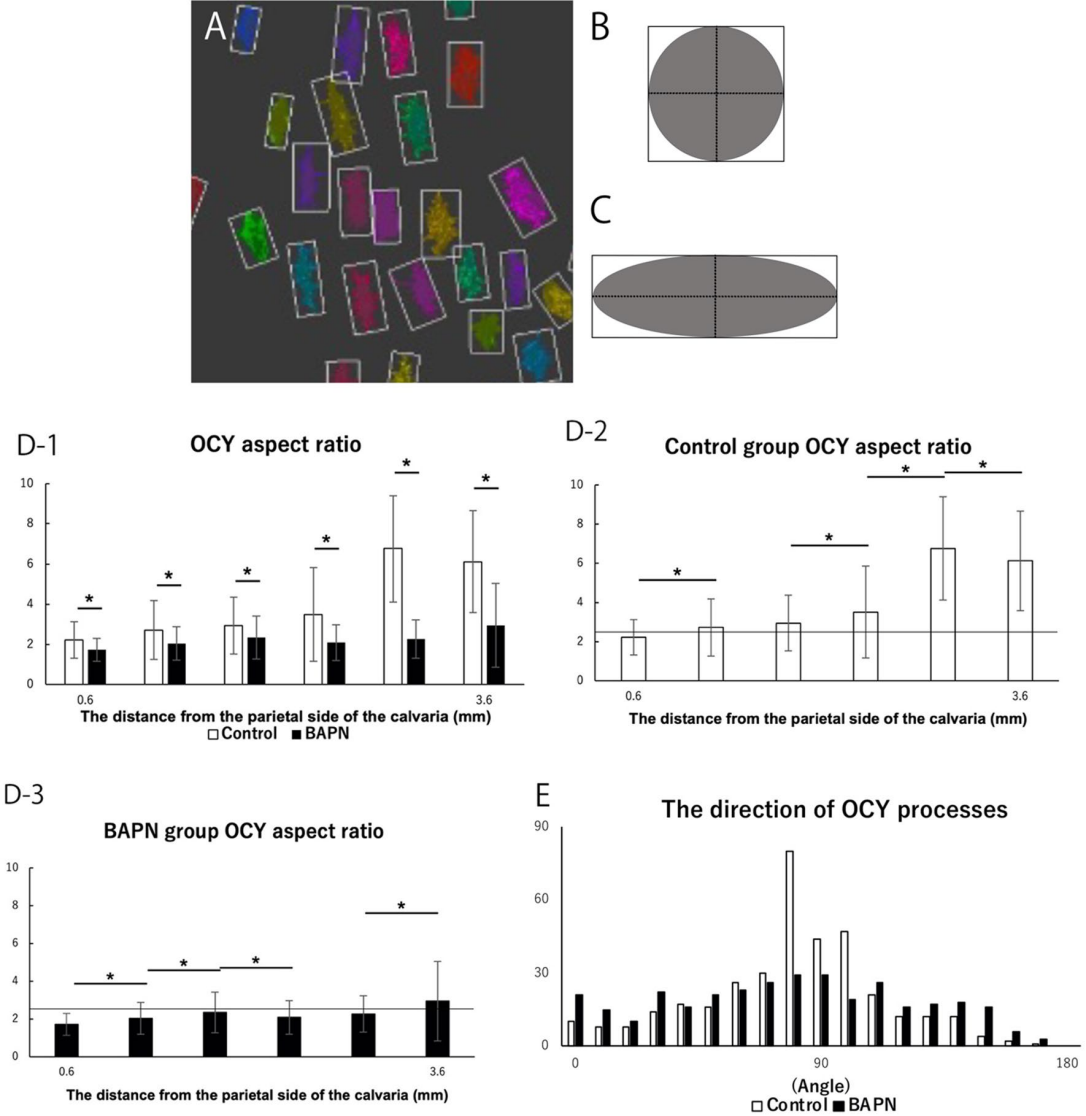
Analysis of osteocyte morphometry. (**A**) The osteocytes of Fig. 4C were recognized as rectangles. (**B**) Schematic diagram of a cell with aspect ratio of 1.0. (**C**) Schematic diagram of a cell with aspect ratio of 3.0. (**D-1**): Comparison of osteocyte morphometry between the control group and BAPN group in the calvaria from the parietal side to the orbital side. The horizontal axis of the graph shows the distance from the parietal side. The histogram shows the mean values of each aspect ratio of the osteocytes, and error bars represent the standard deviation. Asterisk indicates a statistically significant difference (*P* < 0.05). (**D-2**) Comparison of osteocyte morphometry in the control group in the calvaria from the parietal side to the orbital side. The horizontal axis of the graph shows the distance from the parietal side. The histogram shows the mean values of the aspect ratio of the osteocytes in the control group, and error bars represent the standard deviation. Asterisk indicates a significant difference (*P* < 0.05). The horizontal black line shows an aspect ratio of 2.5. (**D-3**) Comparison of osteocyte morphometry in the BAPN group in the calvaria from the parietal side to the orbital side. The horizontal axis of the graph shows the distance from the parietal side. The histogram shows the mean values of the aspect ratio of the osteocytes in the BAPN group, and error bars represent the standard deviation. Asterisk indicates a significant difference (*P* < 0.05). The horizontal black line shows an aspect ratio of 2.5. (**E**) The analysis of the direction of the osteocyte processes. The histogram shows the number of osteocyte processes at each angle.

Osteocyte morphometry was also conducted with the Amira software program. The morphometry method is mentioned in Materials and Methods section (Fig. 5A–C). The aspect ratio of osteocytes of the control group was significantly larger than that of the BAPN group (Fig. 5D-1). In both groups, the aspect ratio of osteocytes became large as they approached the orbital side from the parietal side of the chick calvaria (Fig. 5D-2 and D-3). The osteocyte shape of the control group was sphere shape in the area up to 0.6 mm from the parietal side, and spindle shape from that area toward the orbital side. On the other hand, the osteocyte shape of the BAPN group was sphere shape in the area up to 3.0 mm from the parietal side, and spindle shape in other area.

Osteocytes were randomly extracted, and the direction of the osteocyte processes was analyzed. The osteocyte processes of the control group were vertically elongated to the long axis of the cell body; on the other hand, the osteocyte processes of the BAPN group were radially elongated (Fig. 5E).

## Discussion

The three-dimensional relationship between collagen fibers and embedding late osteoblast processes was observed using orthogonal FIB–SEM (Fig. 1A–C). We also observed that collagen fibrils were produced from osteoblasts. In addition, the embedding late osteoblast processes tended to avoid bundled collagen fibrils as they elongated into newly formed bone matrix. This was found more clearly by reconstructing the serial SEM images three-dimensionally with the Amira software program (Fig. 1D and Supplementary Video 1).

As mentioned in Introduction section, the maturation process of the bone matrix during bone modeling is the production of collagen fibrils by osteoblasts, the orientation of collagen fibrils, the formation of collagen fibers by bundling collagen fibrils, the calcification of collagen fibers, and the formation of osteocytes by embedding osteoblasts into the calcified bone matrix^13,14^. It has been reported that the shape of the intracanalicular surface is dependent on the running of collagen fibrils^15^. Therefore, based on the three-dimensional images acquired in this study, it is strongly suggested that there is a positive relationship between osteocyte network formation and bundling of collagen fibrils.

To further investigate the influence of the bundling of collagen fibrils on osteocyte network formation, cellular network formation was observed when collagen fiber formation was inhibited. BAPN was used to inhibit collagen fiber formation.

Collagen fibrils need to be normally formed in order to form collagen fibers. Cross-linking is essential for collagen fibril formation, and LOX is essential for this cross-linking^19,20^. Therefore, collagen fiber formation was inhibited using BAPN, which is an LOX inhibitor^20,21^. Evaluation of osteocyte viability after BAPN injection was performed with calcein-AM. As a result, BAPN did not affect osteocyte viability in this study (Fig. 2).

SHG imaging was conducted to examine the differences in collagen fiber formation between the control and BAPN groups. SHG imaging makes it possible to observe noncentrosymmetric molecular assemblies (e.g., collagen fibers) in bone tissues without staining^23^. The acquired signal reflects the localization and amount of fibrous structures of the collagen molecule^24^. As a result, the continuity of the collagen fibers in the BAPN group was confirmed to be significantly poorer in comparison to the control group (Fig. 3).

After confirming that BAPN inhibits collagen fiber formation without affecting the osteocyte viability, we observed the change in the osteocyte network. CLS was used instead of electron microscopy (e.g., orthogonal FIB–SEM). This is because electron microscopy is not suitable for observation of the cellular network due to the small observation range. The usefulness of CLS in cellular network observation is certain^12,25–27^. Calvaria specimens were observed from the parietal side to the orbital side using CLS. A significant difference was not recognized between the total number and centroid distance of osteocytes in the control and BAPN groups (Fig. 4E and F). BAPN did not affect osteocyte differentiation.

Then, osteocyte morphometry was performed. When we analyzed osteocytes of both groups from the parietal side to the orbital side, the aspect ratio of the osteocytes of the control group was significantly larger than that of the BAPN group in all areas (Fig. 5D-1). In both groups, the aspect ratio of osteocytes became large as they approached the orbital side from the parietal side of the chick calvaria (Fig. 5D-2 and D-3). The osteocyte shape of the control group was spindle shape in most areas observed, on the other hand, the osteocyte shape of the BAPN group was sphere shape.

When the direction of the osteocyte processes was analyzed, the osteocyte processes of the control group were elongated vertically to the long axis of the cell body; on the other hand, those of the BAPN group were elongated radially (Fig. 5E).

During bone modeling, the collagen fibrils released from osteoblasts run in various directions, but their orientation gradually aligns with the longitudinal axis of the trabecular bone^18^. Early osteocytes are surrounded by collagen fibers. Therefore, it is thought that osteocytes became spindle shaped during trabecular bone growth by aligning the collagen fibers that surround the osteocytes and the growth of trabecular bone. As the osteocytes become spindle shaped and their long axis becomes clear, it is thought that the osteocyte processes elongate vertically to their long axis. In this study, we hypothesized that the decrease in continuity of collagen fibers induced by BAPN was one of the reasons for the change in the shape of the osteocytes.

As bone matures, the arrangement of osteocytes and osteocyte processes has been shown to become very regular, since osteocyte shapes become spindle shaped and their processes elongate vertically to their long axis^28^. It has also been shown that osteocyte processes form cellular networks through gap junctions and this gap junctional intercellular communication (GJIC) play various roles^29–31^. We previously reported that osteocytes changes from spherical to spindle-shaped in embryonic chick calvaria as the osteocytes differentiate. In addition, we also reported that young sphere-shaped osteocytes have a greater GJIC capacity than mature spindle-shaped osteocytes in these calvaria^32^. Therefore, the osteocytes in the BAPN group are considered to be young sphere-shaped osteocytes, and those in the control group are considered to be mature spindle-shaped osteocytes as shown in Fig. 5D. Therefore, it is suggested that osteocyte GJIC capacity of the control group and the BAPN group may be different.

Furthermore, many papers have reported that different osteocyte shapes may have different mechanosensitivity to the same mechanical signal^33,34^. Therefore, it is thought that these osteocytes shape variation of the BAPN group may have an effect on the osteocyte function other than GJIC. It has also been suggested that there is a correlation between the orientation of collagen fibers and lacunae^35,36^; therefore, collagen fibers and osteocytes may be related to each other. In the future, the relationship between collagen fiber formation and the osteocyte network will be discussed more deeply by observing changes in the osteocyte function and gap junctions with the inhibition of collagen fiber formation. In addition, the possibility of collagen fiber-led bone formation will also be discussed, that is, bone matrix-led bone formation, not cell-led bone formation.

## Materials and methods

The use of animals and all animal procedures in this study were approved by the institutional ethics committee on animal research at Okayama University (study number: OKU-2016141, OKU-2019022), and all efforts were made to minimize suffering animals. This study was performed in accordance with the NIH Guide for the Care and Use of Laboratory Animals and ARRIVE guidelines.

### Sample preparation and three-dimensional observation

Sample preparation and three-dimensional observation of the bone by orthogonal FIB–SEM were performed as previously reported^18^. In brief, bone samples were taken from 16-day-old chick embryonic calvaria. The samples were subjected to electron staining, dehydrated, replaced with acetone, embedded in epoxy resin and polymerized in an incubator at 60 °C for 2 days. The embedded samples were polished so that the long axis of the samples and the longitudinal axis of the trabecular bone were parallel, and the observation area was brought to the edge of the sample.

Orthogonal FIB–SEM was performed using a system owned by the National Institute for Materials Science (SMF-1000; Hitachi High-Tech Corporation, Tokyo, Japan). The data of a 25 × 25 × 25-µm region of 25-nm voxels were obtained by setting the sectioning pitch of the FIB to 25 nm and obtaining 1000 SEM images.

The sectional SEM images were three-dimensionally reconstructed using the Amira software program (Zuse Institute Berlin, Berlin, Germany).

### Inhibition of collagen fiber formation by BAPN

BAPN was used to inhibit the bundling of collagen fibrils (collagen fibers). To observe the injection of BAPN into embryonic chicks using stereoscopic microscopy, BAPN solution in PBS was mixed with alizarin red. BAPN was injected into 16-day-old embryonic chicks. Then, 19-day-old embryonic chick calvaria were collected. Based on a previous report, BAPN was injected into the embryonic chick at 350 ml/kg of body weight^37^. Since the body weight of 16-day-old embryonic chicks was approximately 20 g, 0.001% alizarin red solution containing 7 mg BAPN was warmed to 37 °C, and 0.1 ml per embryonic chick was injected by partly breaking the shell. A control group was injected with 0.1 ml of 0.001% alizarin red solution. After injecting the solutions, the egg shell was protected by cellophane film for three days. Three embryonic chicks were used in control group and BAPN group respectively in each experiment.

### Evaluation of osteocyte viability in intact calvarial explants

We used calcein-AM (Molecular Probes, Oregon, USA) to verify the cell viability after BAPN treatment. Calcein-AM is a membrane-permeable dye that cleaved by esterases to calcein within living cells, making it a vitality marker. The dye uptake assay for the estimation of cell viability in calvarial explants was previously described^38^. In brief, 19-day embryonic chick calvarial bone explants were loaded with 5.0 µM calcein-AM for 15 min at room temperature and then subsequently incubated for 30 min in α-MEM with 2% FBS at 37 °C. The total number of analyzed lacunae was 475 in the control group and 426 in the BAPN group. Unpaired t test was used for comparisons of osteocyte viability between the control and BAPN groups. A *P*-value less than 0.05 was considered statistically significant.

### Comparison of collagen fiber formation between the control group and BAPN group

SHG images of chick calvaria were obtained using an upright multiphoton excited microscopy system (A1R MP, Nikon Corporation, Japan) at the Imaging Core Facility of the Institute of Medical Science, the University of Tokyo, as previously reported^39^. The detailed specifications of the microscope system are described elsewhere^23,24,40^. Three-dimensional images were acquired by taking 30 optical slices with an image size of 512 × 512 pixels, a pixel size of 2.49 µm, and a step size of 3 µm. Image processing, including three-dimensional projection and three-dimensional rendering, was performed using the NIS-Elements AR imaging software program (Nikon). Binarization of collagen fibers was interactively conducted using the fluorescence intensity threshold and object size. The recognition of collagen fibers was set as threshold 1000 and size > 15.0 µm. The analysis of collagen fiber formation was performed by a quantitative analysis of the geometric parameters of the collagen fiber binarized images in terms of number, length, outer perimeter, area, and width. Unpaired t tests were used for comparisons between the control and BAPN groups. A *P*-value less than 0.05 was considered statistically significant.

### Comparison of morphological changes between the control group and the BAPN group

Collected calvaria were rinsed and fixed with 3% paraformaldehyde in 60 mmol/L piperazine-N′,N′-bis[2-ethane-sulfonic acid], 25 mmol/L N-[2-hydroxyethyl]piperazine-N′-[2-ethanesulfonic acid], 10 mmol/L ethylene glycol-bis[2-amino-ethyl ether]-N,N,N′,N′-tetraacetic acid, and 2 mmol/L magnesium chloride, pH 6.9 (PHEM buffer) for 10 min at 37 °C. After rinsing, specimens were permeabilized by incubation in 0.1% Triton X-100 in PHEM for 15 min. Specimens were then rinsed and stained for 24 h at 4 °C with Alexa Fluor 488 phalloidin in PBS containing 1% BSA. After rinsing with PBS, the samples were embedded in fluorescence mounting medium (Dako, Carpinteria, CA, USA) containing 1 mg/ml p-phenylenediamine dihydrochloride (Sigma, St. Louis, MO, USA) and then viewed immediately^12^.

Confocal laser scanning microscopy was used to observe the morphological changes in the osteocytes. The CLS conditions were described in a previously reported paper^12^. Confocal images were taken with a 0.5 µm step size and processed four times with Kalman averaging. The images had a frame size of 318.98 × 318.98 µm. All images were analyzed using the Amira software program. The total number and centroid distance of osteocytes were compared between the control and BAPN groups.

For the morphometric classification of osteocytes, first, the aspect ratio of the osteocytes was calculated. The morphometry was performed as follows: The osteocytes were extracted from the CLS images automatically by the Amira software program. They were reconstructed as rectangles (Fig. 5A), and the aspect ratio of the rectangles was measured. If the ratio was < 2.5, we judged that the osteocyte shape was sphere (Fig. 5B). If the ratio was > 2.5, we judged that the osteocyte shape was spindle (Fig. 5C)^32^. A total of 811 osteocytes were analyzed in the control group and 775 were analyzed in the BAPN group. Unpaired t tests were used for comparisons between the control and BAPN groups (Figs. 4E,F and 5D-1). Paired t tests were used for comparisons between the control groups and between the BAPN groups (Fig. 5D-2 and D-3). A *P*-value less than 0.05 was considered statistically significant.

Subsequently, the direction of the osteocyte processes was measured. A total of 18 osteocytes were analyzed in the control group and 20 were analyzed in the BAPN group. The total number of measured osteocyte processes was 364 in the control group and 333 in the BAPN group.

### Supplementary Information

Supplementary Legends.

Supplementary Video 1.

Supplementary Video 2.

## Data Availability

The datasets generated and/or analysed during the current study are available from the corresponding author on reasonable request.
