# Supplementary material for: Bundling of collagen fibrils influences osteocyte network formation during bone modeling
Source: Sci Rep. 2023 Dec 12;13:22028. doi: 10.1038/s41598-023-48786-y (PMC10716128; doi:10.1038/s41598-023-48786-y)
Supplement: Supplementary file 1 — Supplementary Legends. [file 41598_2023_48786_MOESM1_ESM.pdf]

Title

Bundling of collagen fibrils influences osteocyte network formation during bone modeling

Authors

Mana Hashimoto[a], Haruka Takahashi[a], Kaori Tabata-Okubo[b], Noriyuki Nagaoka[c], Kazuaki Tokunaga[d], Haruka Matsumori[d], Yoshihito Ishihara[a], Masaru Kaku[e], Tadahiro Iimura[f], Toru Hara[g], Hiroshi Kamioka[a※]

Affiliations

[a] Department of Orthodontics, Graduate School of Medicine, Dentistry and Pharmaceutical Sciences, Okayama University, 2-5-1, Shikata-cho, Kita-Ku, Okayama, Okayama, 700-8525, Japan

[b] Department of Orthodontics, Okayama University Hospital, 2-5-1, Shikata-cho, Kita-Ku, Okayama, Okayama, 700-8525, Japan

[c] Advanced Research Center for Oral and Craniofacial Sciences, Okayama University Dental School, 2-5-1, Shikata-cho, Kita-Ku, Okayama, Okayama, 700-8525, Japan

[d] Nikon Corporation, 2-15-3 Konan, Minato-Ku, Tokyo, 108-6290, Japan

[e] Division of Bio-prosthodontics, Faculty of Dentistry & Graduate School of Medical and Dental Sciences, Niigata University, 2-5274 Gakkocho-dori, Chuo-ku, Niigata, Niigata, 951-8514, Japan

[f] Department of Pharmacology, Faculty and Graduate School of Dental Medicine, Hokkaido University, N13 W7, Kita-Ku, Sapporo, Hokkaido, 060-8586, Japan

[g] Research Center for Structural Materials, National Institute for Materials Science, 1-2-1, Sengen, Tsukuba, Ibaraki, 305-0047, Japan

Supplementary video 1：Video of serial SEM images

The video shows a three-dimensional reconstruction of serial SEM images.

Supplementary video 2：Video of osteocytes and bundled collagen fibrils

The video shows osteocytes and bundled collagen fibrils which were extracted three-dimensionally.
